# Mitochondria: Impaired mitochondrial translation in human disease

**DOI:** 10.1016/j.biocel.2013.12.011

**Published:** 2014-03

**Authors:** Veronika Boczonadi, Rita Horvath

**Affiliations:** Institute of Genetic Medicine, Wellcome Trust Centre for Mitochondrial Research, Newcastle University, Newcastle upon Tyne, UK

**Keywords:** Mitochondrial respiratory chain, Mitochondrial translation, Human mitochondrial disease, Tissue specific presentation, Cytosolic translation

## Abstract

Defects of the mitochondrial protein synthesis cause a subgroup of mitochondrial diseases, which are usually associated with decreased activities of multiple respiratory chain (RC) enzymes. The clinical presentations of these disorders are often disabling, progressive or fatal, affecting the brain, liver, skeletal muscle, heart and other organs. Currently there are no effective cures for these disorders and treatment is at best symptomatic. The diagnosis in patients with multiple respiratory chain complex defects is particularly difficult because of the massive number of nuclear genes potentially involved in intra-mitochondrial protein synthesis. Many of these genes are not yet linked to human disease. Whole exome sequencing rapidly changed the diagnosis of these patients by identifying the primary defect in DNA, and preventing the need for invasive and complex biochemical testing. Better understanding of the mitochondrial protein synthesis apparatus will help us to explore disease mechanisms and will provide clues for developing novel therapies.

## Highlights

• We present an overview on mitochondrial protein synthesis.
• We summarise nuclear factors involved in mitochondrial protein synthesis.
• Tissue specific presentations highlight molecular mechanisms.
• Altered modification of mt-tRNAs is a frequent cause of tissue specific presentations.
• Interaction between cytosolic and mitochondrial translation needs further investigations.

**Organelle facts**

• Mitochondrial protein synthesis requires several mitochondrial and nuclear-encoded factors for optimal translation.
• The clinical presentation of diseases due to defective mitochondrial protein synthesis is very variable and tissue specific presentations are common.
• The reasons behind the tissue specificity are largely unknown.
• Besides mitochondrial tRNA mutations and mtDNA deletions or depletion, autosomal recessive mutations have been reported in genes encoding ribosomal proteins, ribosome assembly proteins, mitochondrial aminoacyl-tRNA synthetases, tRNA modifying enzymes and initiation, elongation and termination factors of translation.
• Frequent and clinically recognisable genetic causes of human diseases due to impaired mitochondrial translation are caused by mutations in mitochondrial tRNA synthetase and tRNA modifying genes.
• The potential interaction between cytosolic and mitochondrial translation requires further investigations.

## 1 Introduction

Mitochondrial diseases affect at least 1 in 5000 of the population and produce diverse clinical phenotypes often presented as multi-systemic disorders (DiMauro et al., 2013; Ylikallio and Suomalainen, 2012; Vafai and Mootha, 2012). In addition to the nucleus, human cells also harbour DNA in the mitochondria (mtDNA), which is essential for cell viability (Tuppen et al., 2010). This small (16.5 kb) genome is found in multiple copies in mitochondria, the subcellular organelles that often constitute more than 20% of the total cell volume. OXPHOS (oxidative phosphorylation) is responsible for the production of ATP by generating a proton gradient across the inner membrane of the mitochondria which is used by the mammalian cells (Greaves et al., 2012). The mitochondrial OXPHOS system comprises around 150 different proteins out of which only 13 polypeptide subunits are encoded by the mtDNA. In addition, the mtDNA encodes the small and large rRNAs, and 22 distinct mitochondrial tRNAs that are necessary for the translation of only the mitochondrial-encoded proteins (Smits et al., 2010; Rötig, 2011; Chrzanowska-Lightowlers et al., 2011). The nuclear-encoded subunits of the respiratory chain (RC) complexes as well as proteins that are inevitable for normal mitochondrial protein synthesis (such as OXPHOS assembly, mtDNA metabolism and maintenance, mitochondrial cofactor biosynthesis, mitoribosomal subunits and assembly factors, regulators of mitochondrial expression and translation, etc.) are encoded by the nuclear genome (nDNA) and synthetised in the cytosol before transported into the organelle (Vafai and Mootha, 2012). The mitochondrial ribosomal proteins assemble with mitochondrial ribosomes 12S rRNA and 16S rRNA to form the mitochondrial ribosome (Pietromonaco et al., 1991). Lately it was also reported that import of 5S rRNA is also transported to the mitochondria being an essential component of the mitochondrial ribosomes (Smirnov et al., 2011).

## 2 Organelle function

**Fig. 1:**
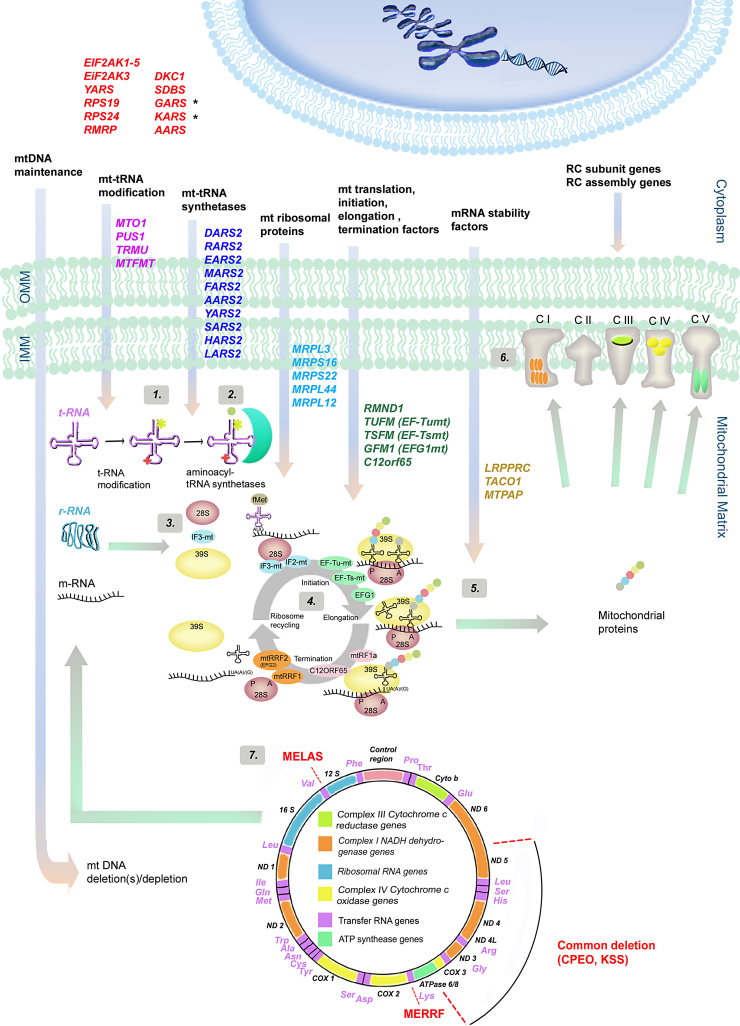
Schematic overview of human genes involved in mitochondrial protein synthesis defects. Prior to mitochondrial protein synthesis the mtDNA needs to be maintained and correctly replicated and transcribed. Mutations within nDNA-encoded genes responsible for these functions lead to mtDNA deletion(s) and depletion. Several other proteins also have to be imported into the mitochondria for accurate mitochondrial translation processes. These nuclear encoded genes are categorised into different groups based on their role in the translational machinery. The first group of genes (highlighted in red) are the genes that are involved in cytosolic translation. Some of these nuclear genes (*GARS*, *KARS* (*marked with black stars*)) are transported to the mitochondria and also function as mitochondrial aminoacyl t-RNA synthetase (ARS). Nuclear genes involved in mt-tRNA modification are: *MTO1*, *PUS1*, *TRMU* and *MTFMT* (highlighted in purple) (the functions of the genes are shown and labelled as (**1**). Up to date 10 mitochondrial ARSs have been found to cause translational deficiencies in humans. These genes are *DARS2*, *RARS2*, *EARS2*, *MARS2*, *FARS2*, *AARS2*, *YARS2*, *SARS2*, *HARS2* and *LARS2* (highlighted in dark blue) (**2**). Nuclear genes encoding for ribosomal proteins and involved in impaired mitochondrial translation are *MRPL3*, *MRPS16*, *MRPS22*, *MRPL12* and *MRPL44* (light blue) (**3**). Genes represented in green are responsible for the mitochondrial translation steps: initiation, elongation and termination (**4**): *RMND1*, *TUFM*, *TSFM*, *GFM1*, *C12orf65*. Nuclear genes such as translational activators and mRNA stability factors (*LRPPRC*, *TACO1* and *MTPAP*) also involved in impaired mitochondrial protein synthesis (**5**). For the formation of the respiratory chain (RC) complexes both nuclear and mitochondrial DNA are required. nDNA-encoded RC subunit genes and RC assembly factors need to be synthetised, transported to the mitochondrial matrix and assembled into functional enzyme complexes with the 13 mDNA-encoded proteins. These 13 proteins are represented within each complex (CI: ND1, ND2, ND3, ND 4L, ND4, ND5, ND6; CIII: CYTB; CIV: COX1, COX2, COX3; CV: ATPase6, ATPase8) (**6**). Schematic drawing of the mitochondrial genome (**7**) showing the 13 proteins, 2 r-RNA, 22 t-RNA regions and the control region. Mutations for MELAS, MERRF and for common deletions are shown in the diagram. Associated clinical phenotypes with the gene mutations mentioned above are detailed in Table 1.

The components responsible for the proper mitochondrial translation are different from their cytosolic counterparts and they are more related to those of bacteria however the mechanisms of the translation follows the same major steps: initiation, elongation, termination and recycling of the ribosome (Christian and Spremulli, 2012) (Fig. 1).

### 2.1 Initiation

The process of mitochondrial translation starts with the formation of the initiation complex. The separation of the two mitochondrial ribosomal subunits (28S and 39S) (Kuzmenko et al., 2013) allow this complex to be formed which consists of the 28S subunit, mRNA and fMET-tRNA and IF2/3_mt_ (Koc and Spremulli, 2002; Christian and Spremulli, 2009). This is followed by the entrance of the mRNA into the IF3_mt_:28S subunit complex. IF3_mt_ is thought to support the correct position of mRNA to bind the small subunit at the peptidyl (P) site of the mitoribosome. When the appropriate start codon is present, the formylmethionin-tRNA can bind to the first codon with the help of IF2_mt_. The association of the mitoribosome stimulates the release of the initiation factors and the elongation on the 55S ribosome commence. MTFMT is critical for efficient human mitochondrial translation and reveal a human disorder of Met-tRNA(Met) formylation (Tucker et al., 2011; Neeve et al., 2013).

### 2.2 Elongation

To coordinate accurate and specific codon anti-codon pairing, mitochondrial elongation factor (EF-Tu_mt_-GTP) and an amino-acylated tRNA arrives to the A-site of the mitoribosome. Upon correct codon-anticodon pairing the EF-Tu_mt_-GDP leave the mitoribosome and the aminoacyl-tRNA moves into the P site where peptide bond formation is catalysed extending the growing polypeptide chain. EF-Ts_mt_ plays a role as a nucleotide exchange factor and converts EF-Tu_mt_ to an active form (EF-Tu_mt_-GTP). The GTP bound EFG1_mt_ catalyses the translocation of the ribosome during the A and P site tRNAs move to the P and exit (E) sites of the mitoribosome. This elongation step repeats itself until the stop codon (UAA, UAG, AGA or AGG) is encountered in the A-site. Mutation of the mitochondrial elongation factors typically associated with encephalopathy and other organ involvement (liver, heart). Clinical symptoms are present in early infancy and the affected children die early (Valente et al., 2007; Smeitink et al., 2006; Coenen et al., 2004; Smits et al., 2011a,b).

### 2.3 Termination

The mitochondrial release factor (mtRF1a) recognises the stop codon and binds to the mitoribosome, induces hydrolysis of the peptidyl-tRNA bond in the A-site releasing the mature protein from the site. Other termination release factors such as mtRF1, C12orf65 and ICT1 are also thought to play an essential role in the termination (Richter et al., 2010). As a last step mitochondrial recycling factors (mtRRF1 and mtRRF2) translocate to the A-site to induce the release of the mRNA (Chrzanowska-Lightowlers et al., 2011). Up to date only the *C12orf65* has been identified as a disease causing gene. Affected patients develop optic atrophy and ophthalmoplegia with Leigh syndrome (Antonicka et al., 2010).

### 2.4 Regulatory mechanisms

The expression of mitochondrial proteins is regulated by their own translational activators that bind mitochondrial mRNAs usually to their 5‘-untranslated regions, and each mitochondrial mRNA has its own translational activator(s), which has been first shown in yeast (Herrmann et al., 2013). Recent studies showed that these translational activators can be part of a feedback control loops which only permit translation if the downstream assembly of nascent translation products can occur (Herrmann et al., 2013). Recently mutations in nuclear-encoded translational activators of mitochondrial proteins such as *TACO1* were also implicated in human disease (Weraarpachai et al., 2009). A regulatory role of aminoacyl-tRNA synthetases has been suggested in both cytosolic and mitochondrial translation (Yao and Fox, 2013) and other factors, such as MTERF3 has been implicated to coordinate crosstalk between transcription and translation for the regulation of mammalian mtDNA (Wredenberg et al., 2013).

## 3 Cell Physiology

Impaired mitochondrial translation usually results in severe combined respiratory chain dysfunction through deficient function of all mtDNA-encoded proteins however some nuclear genes have been shown to alter the translation of single mitochondrial-encoded proteins. The defective mitochondrial proteins lead to deficient ATP production, and cellular energy deficit.

Human cells contain 17 cytoplasmic ARS polypeptides, including the bifunctional glutamyl-prolyl-tRNA synthetase (EPRS), and 18 mitochondrial ARS2 enzymes. Three ARS genes encode proteins with dual localisation, present both in the cytoplasm and mitochondria (GARS, KARS, QARS), and the transport of the mitochondrial isoforms is ensured by a mitochondrial targeting signal.

Beside disorders due to impaired mitochondrial translation, several human disorders are caused by altered cytosolic translation (Yao and Fox, 2013). Interestingly these diseases also lead to tissue specific clinical presentations mainly affecting brain, spinal cord and peripheral neurons, illustrated by clinical presentations such as Charcot–Marie–Tooth disease (CMT), distal hereditary motor neuropathies (dHMN) or leukoencephalopathy with vanishing white matter (VWM). Further implications of altered translation are highlighted by variable and complex clinical presentations, including diseases of eye, cartilage, skin, hair and even cancer (Table 1).

## 4 Organelle pathology

As it was predicted, mutations in the nuclear genes coding for various components of the translation machinery could give rise to a wide spectrum of diseases and phenotypes (Chrzanowska-Lightowlers et al., 2011) (Table 1). Mitochondrial protein synthesis requires several nuclear-encoded factors, such as ribosomal proteins, ribosome assembly proteins, aminoacyl-tRNA synthetases, tRNA modifying enzymes and initiation, elongation and termination factors of translation (Rötig, 2011) (Fig. 1). Autosomal recessive mutations have been reported in several of these factors in association with variable clinical presentations (Chrzanowska-Lightowlers et al., 2011). Here we note, that disorders of mitochondrial protein synthesis usually result in combined RC deficiencies and associated with abnormal mitochondria (ragged red fibres, COX negative fibres) on histology. However a defect of translation activation factors or post-transcriptional regulators of mammalian mtDNA expression may cause impairment in the stability of certain mitochondrial transcripts, as reported in patients with *TACO1* and *LRPPRC* deficiency, respectively (Weraarpachai et al., 2009; Harmel et al., 2013). Because these defects appear to affect only a single OXPHOS enzyme (COX), these patients show isolated COX deficiency.

### 4.1 Defective mitochondrial translation due to mtDNA mutations

Frequent causes of impaired mitochondrial translation are mtDNA rearrangements (e.g. Kearns–Sayre syndrome) that affect mitochondrial tRNA and/or rRNA genes or single mt-tRNA point mutations. About half of the mtDNA mutations causing diseases in humans occur in tRNA genes (MELAS, MERRF, etc.) and the heterogeneous clinical manifestations usually reflect variable heteroplasmy (Tuppen et al., 2010; Greaves et al., 2012). Homoplasmic tRNA mutations with variable penetrance and clinical presentations also occur and suggest the role of genetic or epigenetic modifiers in mitochondrial translation (Taylor et al., 2003). Although most of these conditions are progressive and fatal, reversible infantile cytochrome c oxidase deficiency myopathy (or reversible infantile respiratory chain deficiency), due to a homoplasmic mt-tRNA^Glu^ mutation stands out by showing spontaneous recovery (Horvath et al., 2009).

### 4.2 Defective mitochondrial translation due to nuclear gene defects

The currently defined disorders caused by nuclear defects of mitochondrial protein synthesis are usually early-onset, severe, often fatal diseases (Table 1) with extremely variable clinical presentations, and the reason behind is still unclear. Patients with translation elongation factor or mitochondrial ribosomal protein defects had an early age of onset and a severe multisystem disease with symptoms already present at birth or even prenatal in a few cases (Table 1).

The extreme variability and relative strict tissue specificity of the diseases caused by mutations in mitochondrial tRNA synthetase genes illustrate the importance of understanding the factors influencing mitochondrial translation in different tissues.

### 4.3 Neurological presentations

Some genes are selectively important in specific neuronal populations, as exemplified by leukoencephalopathy with brainstem and spinal cord involvement (LBSL) due to mutations in the mitochondrial aspartyl-tRNA synthetase 2 (*DARS2*), or pontocerebellar hypoplasia caused by argynyl tRNA synthetase 2 (*RARS2*) defect (Scheper et al., 2007; Edvardson et al., 2007). Mutations in the glutamyl-tRNA synthetase (*EARS2*) cause early onset severe neurological disease (leukoencephalopathy involving the thalamus and brainstem with high lactate, LTBL) (Steenweg et al., 2012; Ghezzi et al., 2012). MTFMT deficiency leads to (relatively mild) Leigh syndrome with or without optic atrophy (Tucker et al., 2011; Neeve et al., 2013).

### 4.4 Cardiac presentations

Recently autosomal recessive mutations were reported in the *AARS2* and *MTO1* genes in patients with infantile hypertrophic cardiomyopathy. *MTO1* encodes the enzyme that catalyzes the 5-carboxymethylamino-methylation of the wobble position in mt-tRNA^Glu^, mt-tRNA^Gln^ and mt-tRNA^Lys^ (Ghezzi et al., 2012). Patents with clinically indistinguishable clinical presentation of fatal infantile hypertrophic cardiomyopathy had mutations in the mitochondrial alanyl-tRNA synthetase 2 (*AARS2*) gene (Götz et al., 2011).

### 4.5 Hepatic presentations

Autosomal recessive mutations in the tRNA 5-methylaminomethyl-2-thiouridylate methyltransferase (*TRMU*), which is responsible for 2-thiouridylation of the mt-tRNA^Glu^, mt-tRNA^Gln^ and mt-tRNA^Lys^ cause a severe but reversible infantile hepatopathy (Zeharia et al., 2009; Schara et al., 2011; Sasarman et al., 2011). Infants with reversible hepatopathy develop symptoms between 2 and 4 months of age, but if they survive this phase of liver failure, they recover and develop normally (Schara et al., 2011). The disease course and age of manifestation in TRMU deficiency shows remarkable similarities to reversible infantile myopathy and recent studies suggest that infantile cysteine concentrations may be important for the reversibility of both of these diseases (Boczonadi et al., 2013).

### 4.6 Haematological presentations

As a further complication of mt-tRNA synthetase dysfunction, the involvement of blood cells has been implicated by mutations in *PUS1*, resulting in mitochondrial myopathy, lactic acidosis and sideroblastic anaemia (MLASA) (Bykhovskaya et al., 2004). Mitochondrial tyrosyl-tRNA synthetase 2 (*YARS2*) mutations have been also identified in families with MLASA, very similar, but earlier onset compared to the phenotype caused by deficiency of a mt-tRNA modifying enzyme PUS1 (Chrzanowska-Lightowlers et al., 2011).

### 4.7 Other presentations

Other characteristic, rare diseases are HyperUricemia, Pulmonary hypertension, Renal failure and Alkalosis (HUPRA) syndrome, which is caused by mutations in the mitochondrial seryl-tRNA synthetase 2 (*SARS2*) (Belostotsky et al., 2011) and Perrault syndrome, characterised by ovarian dysgenesis and sensorineural hearing loss due to mutations in the mitochondrial histidyl-tRNA synthetase 2 (*HARS2*) (Pierce et al., 2011) and leucyl-tRNA synthetase 2 (*LARS2*) (Pierce et al., 2013).

## 5 Future outlook

Despite the rapid advances in technologies and the growing number of human disease genes and studies on mechanisms of mammalian mitochondrial translation, its regulation still remains largely unexplored. The variety and intriguing tissue and cell-type specific clinical presentations in both mitochondrial and cytosolic translation, and the dual function of some ARS enzymes suggest substantial interaction and overlap between these two protein synthesis pathways, which have not been extensively studied to date. Due to the abundant proteins and factors required for maintaining accurate mitochondrial translation, it is a challenge to identify the genetic defect in all cases. However rapid development of genetic technologies (next generation sequencing) resulted in a dynamic improvement in genetic diagnosis. Although there is a phenotypic diversity in patients with mitochondrial translation deficiencies, we observed some emerging clinical subgroups (Kemp et al., 2011), which recently turned out to be associated with specific genetic defects. In patients with neurological presentation tRNA synthetases or tRNA modifying factors are the most likely cause of disease. *AARS2* and *MTO1* mutations are preferentially associated with cardiomyopathy, mutations in *TRMU* present with infantile, reversible liver failure, *YARS2* and *PUS1* mutations lead to sideroblastic anaemia and myopathy, and *RMND1* deficiency cause deafness, myopathy, renal involvement and a severe biochemical defect (Table 1).

Defining the exact pathomechanisms will suggest new avenues for treatment in these disorders, as it has been recently studied in reversible COX deficiency myopathy and TRMU deficiency (Boczonadi et al., 2013). Downregulation of TRMU that is required for 2-thiouriylation in cells from patient with RIRCD led to a reduction in levels of mt-tRNA^Glu^ thiolation resulting in a defect of mitochondrial protein synthesis. Cysteine is essential for normal thiolation and supplementation of L-cysteine improved mitochondrial gene translation not only in TRMU but also in RIRCD cells. Interesting experimental progresses are being pursued towards gene therapeutic approaches for mitochondrial translational disorders. Engineered human mitochondrial tRNAs and mRNAs – containing RP import sequence – can be efficiently imported into the mitochondria where they restore translation (Wang et al., 2012). The use of exosomes the body's own vehicle mechanism for delivering protein and genetic biomarkers are also promising and new avenues are being identified. Recently it has been shown that mRNAs for most tRNA synthetases can be detected in exosomes (Wang et al., 2013). The detection of mutations in factors involved in mitochondrial translation widens our understanding of mitochondrial disease and highlights basic molecular mechanisms.

## Figures and Tables

**Table 1:** Nuclear DNA mutations involved in impaired mitochondrial translation and associated diseases in human. Sources: OMIM (Online Mendelian Inheritance in Man).

Nuclear genes involved in impaired cytosolic translation					
Gene	Protein	Clinical presentation	Age of onset	OMIM	References
*EIF2AK1-5*	eIF2B subunits α–ɛ	Vanishing white matter; childhood ataxia with central nervous system hypomyelination (chronic progressive, an episodic encephalopathy)	Childhood to adult age	604032	Leegwater et al. (2001); van der Knaap et al. (2002)
*EIF2AK3*	eIF2 α kinase PERK	Wolcott–Rallison syndrome (diabetes mellitus, epiphyseal dysplasia, kidney and liver dysfunction, mental retardation, central hypothyroidism and dysfunction of the exocrine pancreas)	Neonatal or early childhood	604032	Delépine et al. (2000)
*GARS YARS KARS AARS*	Glycyl-tRNA synthetase tyrosyl-tRNA synthetase lysyl-tRNA synthetase alanyl-tRNA synthetase.	Dominant intermediate Charcot–Marie–Tooth type C (slowly progressive mixed demyelinating-axonal neuropathy) or hereditary motor neuropathy	Childhood to adult age	600287 603623 601421 601065	Antonellis et al. (2003)Jordanova et al. (2006)Rossor et al. (review) (2013)
*RPS19 RPS24*	Ribosomal protein S19 ribosomal protein S24	Diamond–Blackfan anaemia (abnormalities of the thumb, short stature, ventricular septal defects, kidney hypoplasia and congenital glaucoma)	From birth	603474 602412	Draptchinskaia et al. (1999); Gazda et al. (2006)
*RMRP*	Mitochondrial RNA proc. endoribonuclease	Cartilage-hair hypoplasia	Neonatal, infantile	157660	Ridanpää et al. (2001)
*DKC1*	Dyskerin	X-linked dyskeratosis congenita (ectodermal abnormalities, bone marrow failure and susceptibility to cancer)	From birth	300126	Heiss et al. (1998)
*SBDS*	Shwachman–Bodian–Diamond syndrome protein	Shwachman–Diamond syndrome (exocrine pancreatic insufficiency, bone marrow dysfunction, skeletal abnormalities and short stature)	From birth	607444	Boocock et al. (2003)

Nuclear genes involved in impaired mitochondrial translation – tRNA-modifying enzymes					
Gene	Protein	Clinical presentation	Age of onset	OMIM	References
*PUS1*	Pseudouridine synthase	Myopathy, lactic acidosis and sideroblastic anaemia (MLASA1)	Early childhood	608109	Bykhovskaya et al. (2004)
*TRMU*	tRNA 5-methylaminome-thyl-2-thiouridylate methyl-transferase	Reversible infantile liver failure	Infantile	613070	Zeharia et al. (2009); Gaignard et al. (2013)Schara et al. (2011);
*MTO1*	Mitoch. translation optimisation 1 homolog	Hypertrophic cardiomyopathy and lactic acidosis	Infantile	614702	Ghezzi et al. (2012)
*MTFMT*	Mitoch. methionyl-tRNA formyltransferase	Leigh syndrome	Early childhood	611766	Tucker et al. (2011)Neeve et al. (2013)

Nuclear genes involved in impaired mitochondrial translation – ribosomal proteins					
Gene	Protein	Clinical presentation	Age onset	OMIM	References
MRPL3	Mitochondrial ribosomal protein L3	Hypertrophic cardiomyopathy and psychomotor retardation	Infantile	614582	Galmiche et al. (2011)
*MRPS16*	Mitochondrial ribosomal protein S16	Corpus callosum agenesia, hypothonia and fatal neonatal lactic acidosis	Neonatal	610498	Miller et al. (2004)
*MRPS22*	Mitochondrial ribosomal protein S22	Cornelia de Lange-like syndrome oedema, cardiomyopathy and tubulopathy	Neonatal	611719	Saada et al. (2007); Smits et al. (2011a,b)
*MRPL12*	Mitochondrial ribosomal protein L12	Growth retardation and neurological deterioration	Neonatal	602375	Serre et al. (2013)
*MRPL44*	Mitochondrial ribosomal protein L44	Hypertrophic cardiomyopathy	Neonatal	611849	Carroll et al. (2013)

Nuclear genes involved in impaired mitochondrial translation – aminoacyl-tRNA synthetases					
Gene	Protein	Clinical presentation	Age onset	OMIM	References
*DARS2*	Aspartyl-tRNA sythetase 2	Leukoencephalopathy with brainstem and spinal cord involvement (LBSL)	Childhood or adulthood	610956	Scheper et al. (2007)
*RARS2*	Arginyl-tRNA synthetase 2	Pontocerebellar hypoplasia type 6 (PCHD-6)	Neonatal	611523	Edvardson et al. (2007)
*EARS2*	Glutamyl-tRNA synthetase 2	Leukoencephalopathy with thalamus and brainstem involvement and high lactate (LTBL)	Infantile	612799	Steenweg et al. (2012)
*MARS2*	Methionyl-tRNA synthetase 2	Autosomal recessive spastic ataxia with leukoencephalopathy	Juvenile or adulthood	609728	Bayat et al. (2012)
*FARS2*	Phenylalanyl-tRNA synthetase 2	Alpers syndrome, encephalopathy, epilepsy, lactic acidosis	Neonatal and infantile	611592	Elo et al. (2012); Shamseldin et al. (2012)
*AARS2*	Alanyl-tRNA synthetase 2	Hypertrophic cardiomyopathy	Infantile	614096	Götz et al. (2011)
*YARS2*	Tyrosyl-tRNA synthetase 2	Myopathy, lactic acidosis, and sideroblastic anaemia (MLASA2)	Infantile	613561	Riley et al. (2010)
*SARS2*	Seryl-tRNA synthetase 2	HUPRA syndrome (hyperuricemia, pulmonary hypertension, renal failure in infancy and alkalosis)	Infantile (4 months)	613845	Belostotsky et al. (2011)
*HARS2*	Histidyl-tRNA synthetase 2	Perrault syndrome (sensorineural deafness, ovarian dysgenesis)	Juvenile or adulthood	600783	Pierce et al. (2011)
*LARS2*	Leucyl-tRNA synthetase 2	Perrault syndrome (sensorineural deafness, ovarian dysgenesis)	Juvenile	604544	Pierce et al. (2013)

Nuclear genes involved in impaired mitochondrial translation – initiation, elongation and termination factors					
Gene	Protein	Clinical presentation	Age onset	OMIM	References
*RMND1*	RMND	Deafness, myopathy, renal involvement and a severe biochemical defect	Neonatal	614917	Garcia-Diaz et al. (2012): Janer et al. (2012)
*TUFM*	Elongation factor Tu, mitochondrial (EF-TU_mt_)	Lactic acidosis, leukoencephalopathy and polymicrogyria	Neonatal	610678	Valente et al. (2007)
*TSFM*	Elongation factor Ts, mitochondrial (EF-Ts_mt_)	Encephalomyopathy, hypertrophic cardiomyopathy	Neonatal	610505	Smeitink et al. (2006)
*GFM1*	Elongation factor G 1, mitochondrial (EFG1_mt_)	Encephalopathy with or without liver involvement	Neonatal	609060	Coenen et al. (2004); Smits et al. (2011a); Valente et al. (2007)
*C12orf65*	Chromosome 12 open reading frame 65	Leigh syndrome, optic atrophy, ophthalmoplegia	Infantile	613559	Antonicka et al. (2010)

Nuclear genes involved in impaired mitochondrial transaltion – translation activators and mRNA stability factors					
Gene	Protein	Clinical presentation	Age onset	OMIM	References
*LRPPRC*	Leucine-rich PPR-motif containing protein	Leigh syndrome French–Canadian variant (LSFC)	Infantile	220111	Mootha et al. (2003)
*TACO1*	Translational activator of cytochrome c oxidase 1	Late-onset Leigh syndrome	Juvenile	612958	Weraarpachai et al. (2009)
*MTPAP*	Mitochondrial poly-A polymerase	Progressive spastic ataxia with optic atrophy	Juvenile (early childhood)	613672	Crosby et al. (2010)
